# Decreased Fitness and Virulence in ST10 *Escherichia coli* Harboring *bla*_NDM-5_ and *mcr-1* against a ST4981 Strain with *bla*_NDM-5_

**DOI:** 10.3389/fcimb.2017.00242

**Published:** 2017-06-08

**Authors:** Yawei Zhang, Kang Liao, Hua Gao, Qi Wang, Xiaojuan Wang, Henan Li, Ruobing Wang, Hui Wang

**Affiliations:** ^1^Department of Clinical Laboratory, Peking University People's HospitalBeijing, China; ^2^Department of Clinical Laboratory, First Affiliated Hospital of Sun Yat-sen UniversityGuangzhou, China

**Keywords:** colistin-resistant *E. coli*, *bla*_NDM-5_, *mcr-1*, fitness, virulence

## Abstract

Although coexistence of *bla*_NDM-5_ and *mcr-1* in *Escherichia coli* has been reported, little is known about the fitness and virulence of such strains. Three carbapenem-resistant *Escherichia coli* (GZ1, GZ2, and GZ3) successively isolated from one patient in 2015 were investigated for microbiological fitness and virulence. GZ1 and GZ2 were also resistant to colistin. To verify the association between plasmids and fitness, growth kinetics of the transconjugants were performed. We also analyzed genomic sequences of GZ2 and GZ3 using PacBio sequencing. GZ1 and GZ2 (ST10) co-harbored *bla*_NDM-5_ and *mcr-1*, while GZ3 (ST4981) carried only *bla*_NDM-5_. GZ3 demonstrated significantly more rapid growth (*P* < 0.001) and overgrew GZ2 with a competitive index of 1.0157 (4 h) and 2.5207 (24 h). Increased resistance to serum killing and mice mortality was also identified in GZ3. While GZ2 had four plasmids (IncI2, IncX3, IncHI2, IncFII), GZ3 possessed one plasmid (IncFII). The genetic contexts of *bla*_NDM-5_ in GZ2 and GZ3 were identical but inserted into different backbones, IncX3 (102,512 bp) and IncFII (91,451 bp), respectively. The growth was not statistically different between the transconjugants with *mcr-1* or *bla*_NDM-5_ plasmid and recipient (*P* = 0.6238). Whole genome sequence analysis revealed that 28 virulence genes were specific to GZ3, potentially contributing to increased virulence of GZ3. Decreased fitness and virulence in a *mcr-1* and *bla*_NDM-5_ co-harboring ST10 *E. coli* was found alongside a ST4981 strain with only *bla*_NDM-5_. Acquisition of *mcr-1* or *bla*_NDM-5_ plasmid did not lead to considerable fitness costs, indicating the potential for dissemination of *mcr-1* and *bla*_NDM-5_ in *Enterobacteriaceae*.

## Introduction

Multidrug-resistant Gram-negative bacteria continue to threaten several aspects of public health due to the availability of limited antimicrobials for treatment, especially for the carbapenem-resistant isolates. Colistin has become the last resort to treat serious infections caused by multidrug-resistant organisms. However, colistin resistance has also been reported in carbapenem-resistant bacteria. Since 2015, plasmid-mediated colistin resistance gene, *mcr-1*, has been reported and identified with increasing incidence in *Enterobacteriaceae* worldwide, isolated from environment, human and animals (Liu, Y. Y. et al., 2016; Nordmann and Poirel, 2016). The co-existence of *mcr-1* and carbapenemase genes, including *bla*_NDM_ and *bla*_KPC_, has already been detected in clinical settings and resulted in a few therapeutic options (Li et al., 2016; Teo et al., 2016). The rapid transmission of *mcr-1* and carbapenemase genes located on mobile genetic elements is of great concern. Successful dissemination of resistance plasmids largely depends on the fitness cost imposed on hosts (Andersson and Hughes, 2010). Therefore, understanding the biological cost of resistance is critical for controlling the dissemination of multi-drug resistant strains.

So far, studies on the biological effects of colistin resistance have mainly focused on *Acinetobacter baumannii* (Hraiech et al., 2013; Beceiro et al., 2014; Wand et al., 2015) with extremely limited data on *Enterobacteriaceae*. The impact of *mcr-1* gene on *in vitro* and *in vivo* fitness is yet to be elucidated. Furthermore, there is a lack of studies concerning the effects of coexistence of carbapenemase and *mcr-1* on fitness and virulence in *Enterobacteriaceae*.

Here, we identified three *Escherichia coli* isolates carrying *bla*_NDM-5_ from a single patient with different sequence type and two of them also harbored *mcr-1*. The purpose of this study was to examine the *in vitro* and *in vivo* fitness, virulence and genetic backgrounds of *bla*_NDM-5_ and *mcr-1* co-producing *Enterobacteriaceae* and verify the association between resistance plasmids and the fitness.

## Materials and methods

### Bacterial isolation and clinical information

Medical records and the patient information were retrospectively reviewed and collected. A 19-year-old female was admitted into a hospital in Guangzhou in April 2015. The patient had a history of pelvic fracture, urethral disruption and pelvic organ injury treated with colostomy and cystostomy. Surgery was performed during her hospitalization. She subsequently presented with fever, fistula of colon and ureter, complex intra-abdominal infections, systemic inflammatory response syndrome and urinary tract infections. From May to July, three carbapenem-resistant *E. coli* (GZ1, GZ2, and GZ3) were successively isolated from abdominal drainage and urine obtained from cystostomy. While she received various antimicrobials, including cefuroxime, gentamycin, metronidazole, ceftazidime, amikacin, meropenem, imipenem, levofloxacin, cefixime, vancomycin and piperacillin–tazobactam before GZ2 was isolated, due to clinical improvement, the treatment was changed to levofloxacin, cefixime and metronidazole after the isolation. The study was approved by the research ethics board at Peking University People's Hospital. Informed consent was not needed as this study was retrospective and participants were anonymized.

### Antimicrobial susceptibility testing, detection of resistance genes and multilocus sequence typing

The minimum inhibitory concentrations (MICs) of the following antimicrobials, including ceftriaxone (Roche, Shanghai, China), ceftazidime, cefoxitin, cefepime, amikacin (National Institutes for Food and Drug Control, Beijing, China), piperacillin–tazobactam, tigecycline (Pfizer, NY, USA), imipenem (MSD, Hangzhou, China), meropenem (Sumitomo Pharmaceuticals, Suzhou, China), ciprofloxacin (Bayer, Leverkusen, Germany) and colistin (Amresco, Solon, USA) were determined by agar dilution method, according to Clinical and Laboratory Standards Institute (CLSI) guidelines (Clinical and Laboratory Standards Institute, 2015). The results were interpreted according to CLSI breakpoints (Clinical and Laboratory Standards Institute, 2016). Polymerase chain reaction (PCR) was used to detect the presence of *mcr-1*, carbapenemase genes and other resistance genes as previously described (Wang et al., 2014; Liu, Y. Y. et al., 2016). Multilocus sequence typing (MLST) was performed as described on the *E. coli* MLST website (http://mlst.warwick.ac.uk/mlst/dbs/Ecoli).

### Growth assay and *In vitro* competition experiment

Fitness was investigated by using growth curve assay and *in vitro* competition experiments (Liu, D. et al., 2016). Three isolates (GZ1, GZ2, and GZ3) cultured overnight in LB broth were diluted to an OD_600_ of 0.01 and grew at 37°C with vigorous aeration (200 rpm). The culture cell density was determined every 0.5 h by measuring the OD_600_.

In the *in vitro* competition assays, GZ2 and GZ3 were separately cultured overnight in LB broth at 37°C. The bacteria were diluted and equivalent numbers of GZ2 and GZ3 were pooled and cultured together at 37°C. At 0, 4, and 24 h, aliquots of the mixed bacteria were diluted with 0.9% saline solution and plated on Mueller–Hinton agar (BD, Sparks MD, USA) plates with or without colistin (4 mg/L). Colony-forming unit (CFU) were counted after 0, 4, and 24 h of incubation at 37°C. Each isolate was tested three times. The competitive index (CI) was determined as follows: CI = (GZ3/GZ2)/(Inoculated GZ3/Inoculated GZ2) as previously described (Liu, D. et al., 2016).

### Serum killing assay

Serum killing assay was conducted to determine the virulence *in vitro* as previously described (Abate et al., 2012). An inoculum of 25 μL prepared from the mid-log phase was diluted by 0.9% saline solution and was added to 75 μL of pooled human sera contained in a 10 × 75 mm Falcon polypropylene tube (BD Biosciences, Franklin Lakes, NJ, USA). Viable counts were checked at 0, 1, 2, and 3 h of incubation at 37°C. The mean results were expressed as percentage of inoculation and a strain was classified as serum sensitive, intermediately sensitive and resistant.

### Mouse lethality assay

To determine *in vivo* virulence, six pathogen-free, 6–8-week-old, male BALB/c mice were used as a sample population for each bacterial concentration. Ten-fold serial dilution of CFU of *E. coli* was made from a starting concentration of 10^9^ CFU/mL to 10^5^ CFU/mL, and BALB/c mice were infected intraperitoneally with 0.1 mL of each concentration. Symptoms and mortality rates were observed for 14 days. Inoculation dose was confirmed on LB agar and survival curves were assessed by Kaplan–Meier analysis. The study was approved by the research ethics board at Peking University People's Hospital.

### Construction of the *E. coli* J53 transconjugants carrying *mcr-1* and *bla*_NDM-5_ plasmids

To exclude the impact on fitness caused by different sequence type, we constructed the same strains carrying different plasmids. Conjugation experiment was performed using GZ2 and GZ3 as the donors and *E. coli* J53 as the recipient according to previous studies (Wang et al., 2014). The donor and the recipient were mixed at a ratio of 1:1 for 24 h. Transconjugants were selected on Chinese Blue lactose agars (OXOID, Basingstoke Hampshire, UK), supplemented with sodium azide (100 mg/L) and imipenem (1 mg/L), sodium azide (100 mg/L) and colistin (4 mg/L) or sodium azide (100 mg/L), colistin (4 mg/L), and imipenem (1 mg/L), respectively. PCR was used to screen for *mcr-1* and *bla*_NDM-5_ as previously described (Wang et al., 2014; Liu, Y. Y. et al., 2016).

### Fitness measurements of the *E. coli* J53 transconjugants

The plasmid profiles of the transconjugants were identified by PCR based on replicons of the major plasmid incompatibility groups as previously described (Carattoli et al., 2005). To verify the association between the resistance plasmids and the fitness, growth kinetics of the transconjugants and the recipient *E. coli* J53 were investigated (Liu, D. et al., 2016). The strains cultured overnight in LB broth were diluted to an OD_600_ of 0.01 and grew at 37°C with vigorous aeration (200 rpm). The culture cell density was determined every 0.5 h by measuring the OD_600_ for 24 h.

### Whole genome sequencing

Whole genome sequencing (WGS) of GZ2 and GZ3 was conducted using a PacBio RS II system (Pacific Biosciences, Menlo Park, USA) with a 10 kb size library and P6/C4 chemistry. *De novo* assembly was performed with SMRTanalysis version 2.3 (Pacific Biosciences). The plasmid sequence was annotated using the online Rapid Annotation Subsequencing Technology (RAST) (http://rast.nmpdr.org/) automatically and BLASTn analysis. Antibiotic resistance genes and plasmid incompatibility groups were analyzed through the website of the Center for Genomic Epidemiology (http://www.genomicepidemiology.org/). Virulence genes were identified using the virulence factor database (http://www.mgc.ac.cn/VFs/) and PathogenFinder (https://cge.cbs.dtu.dk/services/PathogenFinder/). The nucleotide sequences of the genome and plasmids of GZ2 and GZ3 have been submitted to GenBank with accession no. MCRE00000000, CP017980, and CP017981.

### Statistical analysis

Statistical analysis was performed with the software GraphPad Prism version 5 using one-way analysis of variance (ANOVA) followed by Tukey–Kramer tests. *P* < 0.05 was considered to be statistically significant.

## Results

### Antimicrobial susceptibility testing, molecular typing and detection of resistance genes

Results pertaining to susceptibility testing showed that all isolates were resistant to most of the tested antimicrobials including carbapenems. While GZ1 and GZ2 exhibited resistance to colistin (Table 1) and co-harbored *bla*_NDM-5_ and *mcr-1* belonging to ST10, GZ3 (ST4981) carried only *bla*_NDM-5_.

**Table 1:** Characteristics of the isolated *E. coli* strains and its transconjugants.

**Characteristics**	**GZ1**	**GZ2**	**GZ3**	***E. coli*** **J53 transconjugants**			**J53**
				**Tc_pmcr-1GZ2_**	**Tc_pNDM-5GZ2_**	**Tc_pNDM-5GZ3_**	
Specimen type	Abdominal drainage	Urine from cystostomy	Urine from cystostomy	–	–	–	–
Date of collection	5/23/2015	6/19/2015	7/21/2015	–	–	–	–
MLST	ST10	ST10	ST4981	–	–	–	–
Serum killing assay	Sensitive (grade 1)	Sensitive (grade 2)	Intermediate (grade 3)	–	–	–	–
Major resistance genes	*bla*_NDM-5_, *mcr-1*	*bla*_NDM-5_, *mcr-1*	*bla*_NDM-5_	*mcr-1*	*bla*_NDM-5_	*bla*_NDM-5_	–
**Plasmid profiles**							
Number of plasmids	ND^a^	Four	One	Three	Three	One	–
**Plasmid harboring** ***bla***_NDM-5_							
Replicon type	ND^a^	IncX3	IncFII	–	IncX3	IncFII	–
Size	ND^a^	102,512 bp	91,451 bp	–	102,512 bp	91,451 bp	–
Other resistance genes	ND^a^	*aadA2, mph (A), sul1, dfrA12, aac(3)-IId*	*bla*_TEM-1*B*_, *mph (A), erm (B)*	–	*aadA2, mph (A), sul1, dfrA12, aac(3)-IId*	*bla*_TEM-1*B*_, *mph (A), erm (B)*	–
**Plasmid harboring** ***mcr-1***							
Replicon type	ND^a^	IncI2	–	IncI2	–	–	–
Size	ND^a^	59,476 bp	–	59,476 bp	–	–	–
Resistance genes	ND^a^	*mcr-1*	–	*mcr-1*	–	–	–
**Other co-harbored plasmids**							
Replicon type	ND^a^	IncHI2, IncFII	–	IncHI2, IncFII	IncHI2, IncFII	–	–
Size	ND^a^	247,200 bp, 61,231 bp	–	247,200 bp, 61,231 bp	247,200 bp, 61,231 bp	–	–
Resistance genes	ND^a^	*aph (4)-Ia, aac(3)-IVa, aac(6′)Ib-cr, rmtB, aac(3)-IId, aph(3′)-Ia, aadA2, bla*_OXA-1_, *bla*_TEM-1*B*_, *floR, catB3, arr-3, sul2, sul1, dfrA12, mph(A), strB, strA, qnrS1*	–	*aph (4)-Ia, aac(3)-IVa, aac(6′)Ib-cr, rmtB, aac(3)-IId, aph(3′)-Ia, aadA2, bla*_OXA-1_, *bla*_TEM-1*B*_, *floR, catB3, arr-3, sul2, sul1, dfrA12, mph(A), strB, strA, qnrS1*	*aph (4)-Ia, aac(3)-IVa, aac(6′)Ib-cr, rmtB, aac(3)-IId, aph(3′)-Ia, aadA2, bla*_OXA-1_, *bla*_TEM-1*B*_, *floR, catB3, arr-3, sul2, sul1, dfrA12, mph(A), strB, strA, qnrS1*	–	–
**MINIMUM INHIBITORY CONCENTRATION OF ANTIMICROBIALS (μg/mL)**							
Ceftriaxone	>256	>256	256	≤0.016	128	>256	≤0.016
Ceftazidime	>256	>256	>256	0.25	>256	>256	0.125
Cefepime	64	32	16	0.064	8	32	≤0.016
Cefoxitin	>256	>256	>256	8	256	>256	4
Piperacillin-tazobactam	256	>256	128	1	128	256	1
Imipenem	8	16	2	0.25	4	8	0.25
Meropenem	8	16	1	≤0.016	2	4	≤0.016
Amikacin	>256	>256	16	1	1	1	1
Ciprofloxacin	>64	>64	64	0.25	0.25	0.125	≤0.016
Tigecycline^b^	2	1	0.125	0.25	0.25	0.5	0.25
Colistin	8	8	0.25	8	0.125	0.125	0.25

a *ND, Not detected*.

b *The interpretive criteria for tigecycline was based on the breakpoints of FDA*.

### *In vitro* and *In vivo* fitness and virulence of GZ1, GZ2, and GZ3

**Figure 1:**
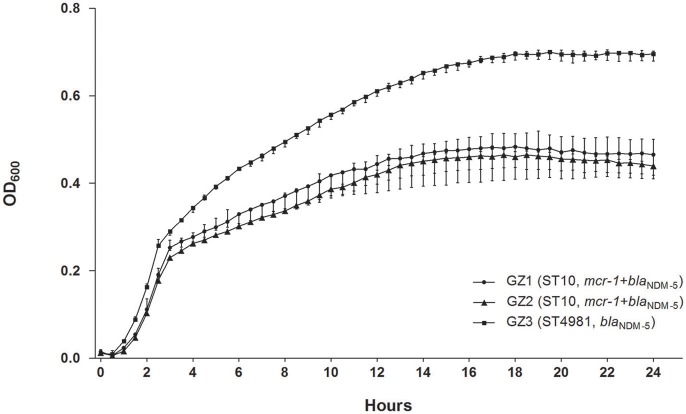
*In vitro* growth of GZ1, GZ2, and GZ3 cultured at 37°C in LB broth. The growth of GZ3 was significantly higher than the other two strains (*P* < 0.001). Data points represent the mean (±standard deviations) of three independent experiments.

The growth of GZ3 was significantly higher than the other two isolates (*P* < 0.001) (Figure 1) and *in vitro* competition experiment revealed that GZ2 was overgrown by GZ3 with a competitive index (CI) of 1.0157 (4 h) and 2.5207 (24 h). GZ3 demonstrated intermediate to serum killing (grade 3), whereas GZ1 and GZ2 displayed sensitive (grade 1 or 2), thus suggesting that in human sera, GZ3 exhibited higher defense against bactericidal activity than GZ1 and GZ2. A significant difference in mice lethality was identified between GZ3 and the other isolates. All mice injected with GZ3 died within the first day at a concentration of 10^9^ CFU/mL, while mice infected with GZ1, GZ2 and the control survived over 14 days.

### Conjugation experiment and biological costs of plasmid carriage

The characteristics of the transconjugants (designated as Tc_pmcr-1GZ2_, Tc_pNDM-5GZ2_, Tc_pNDM-5GZ3_) were summarized in Table 1. In the conjugation experiments, *bla*_NDM-5_ and *mcr-1* could be transferred separately from GZ2 and GZ3 to the recipient *E. coli* J53. No transconjugants co-harboring *mcr-1* and *bla*_NDM-5_ were detected. In addition to plasmids containing *mcr-1* or *bla*_NDM-5_, the plasmid profile of the transconjugants was the same as their corresponding parent strains. According to the plasmid replicon typing results, there were three plasmids in Tc_pmcr-1GZ2_ (IncI2, IncHI2, IncFII), three plasmids in Tc_pNDM-5GZ2_ (IncX3, IncHI2, IncFII) and one plasmid in Tc_pNDM-5GZ3_ (IncFII).

**Figure 2:**
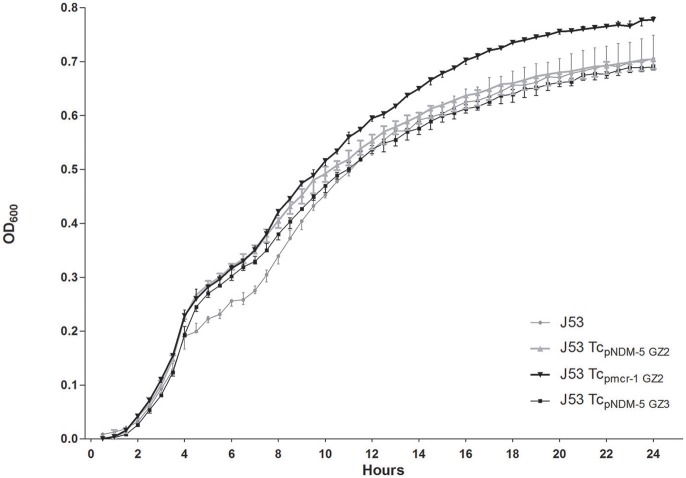
Growth kinetics of *E. coli* J53 and its transconjugants (Tc_pNDM-5GZ2_, Tc_pmcr-1GZ2_ and Tc_pNDM-5GZ3_). Values represent the mean ± standard variations obtained from three independent experiments.

No significant difference in growth was observed in Tc_pmcr-1GZ2_, Tc_pNDM-5GZ2_, Tc_pNDM-5GZ3_, and *E. coli* J53 (*P* = 0.6238) (Figure 2). The results indicated that no considerable fitness costs were identified in carriage of plasmid containing *mcr-1* or *bla*_NDM-5_ compared to the recipient strain, suggesting that resistance was not associated with the decreased fitness of GZ2. Chromosomal characteristics, such as different sequence type (ST10 and ST4981) were considered to be the potential explanations.

### Analysis of virulence genes in GZ2 and GZ3 by WGS

WGS and virulence factor database analysis revealed that GZ2 and GZ3 had 120 and 124 virulence-associated genes, respectively. Compared with GZ2, GZ3 had a higher number of hemolysin genes and 28 specific virulence genes in the genome. These genes were associated with chorismate binding-like protein, iron acquisition, CFA/I fimbria and type III secretion protein (such as *eiv, epr* and *epa*), potentially contributing to the increased virulence in GZ3.

### Sequence analysis of plasmid carrying *mcr-1*

In this study, the plasmids harboring *mcr-1* and *bla*_NDM-5_ were designated as pGZ2-mcr, pGZ2-NDM, and pGZ3-NDM. The plasmid carrying *mcr-1* from GZ2 (named pGZ2-mcr) was 59,476 bp in length, belonging to IncI2 incompatibility group. The results of BLASTn analysis revealed that plasmid pGZ2-mcr with 100% query coverage displayed 99% identity to plasmid pEC13-1 (GenBank accession CP016186.1) and plasmid pBA77-MCR-1 (GenBank accession KX013539.1) which have been isolated from *E. coli* in Malaysia and United Arab Emirates. No other resistance genes were found in pGZ2-mcr.

### Sequence analysis of plasmid containing *bla*_NDM-5_

**Figure 3:**
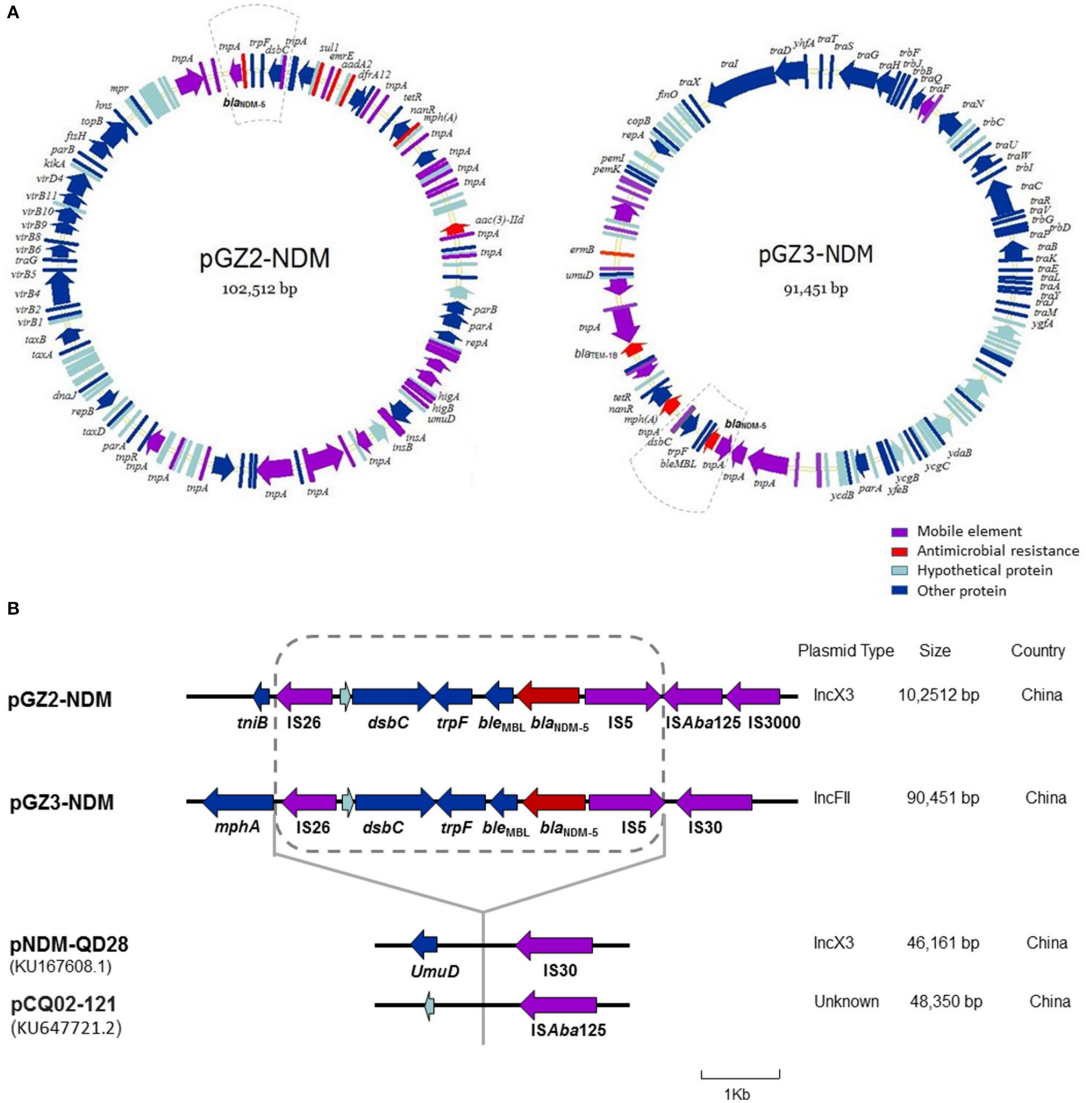
Plasmid analysis of pGZ2-NDM and pGZ3-NDM. Structure map of pGZ2-NDM and pGZ3-NDM **(A)** and comparative analysis of the genetic contexts of *bla*_NDM-5_ in plasmids reported in this study and previously described **(B)**. The gene name is shown next to the corresponding arrow or rod. Genetic regions with dashed lines in pGZ2-NDM and pGZ3-NDM are indicated as *bla*_NDM-5_ region. The genetic environment of *bla*_NDM-5_ (with dashed line) in pGZ2-NDM and pGZ3-NDM was similar as that previously described in pNDM-QD28 (GenBank accession KU167608.1) and pCQ02-121 (GenBank accession KU647721.2) with 100% query coverage and 99% identity. However, the regions containing *bla*_NDM-5_ were inserted into different genes and plasmid backbones.

Sequence analysis showed that the two plasmids carrying *bla*_NDM-5_ were not identical and belonged to different incompatibility group (Figure 3A). While plasmid pGZ2-NDM was 102,512 bp in length and possessed an IncX3-type backbone, pGZ3-NDM (91,451 bp) belonged to IncFII group. Both the plasmids encoded replication, stability, horizontal transfer, antimicrobial resistance, and maintenance functions (Figure 3A). pGZ2-NDM and pGZ3-NDM were found to harbor additional resistance genes, including *aadA2, mph (A), sul1, aac (3)-IId, dfrA12, bla*_TEM−1B_, and *erm (B)* (Table 1). Sequence alignments with BLAST revealed that pGZ2-NDM shared 53% query coverage and 99% identity with plasmid pNDM-ECN49 (GenBank accession KP765744.1), isolated from an *Enterobacter cloacae* strain. The genetic environment of *bla*_NDM-5_ differed between these two plasmids. pGZ3-NDM exhibited more than 70% query coverage and 99% identity with plasmid pHNFP460-1 (GenBank accession KJ020575.1), whereas no *bla*_NDM-5_ gene was found in pHNFP460-1.

Comparative analysis of the genetic contexts of *bla*_NDM-5_ (~4,900 bp) in pGZ2-NDM and pGZ3-NDM were almost identical and were also similar as that previously reported in pNDM-QD28 (GenBank accession KU167608.1) and pCQ02-121 (GenBank accession KU647721.2) with 100% query coverage and 99% identity (Figure 3B). The regions containing *bla*_NDM-5_ were inserted into different genes. Furthermore, these plasmids belonged to different incompatibility groups. Therefore, the results indicated that similar genetic context containing *bla*_NDM-5_ have been inserted into different plasmid backbones, thus forming a “new” plasmid.

## Discussion

To date, there have been several reports on the coexistence of *bla*_NDM-5_ and *mcr-1* in *Enterobacteriaceae* worldwide, but little is known about the fitness and virulence of such strains (Mediavilla et al., 2016; Yang et al., 2016). Several findings are of microbiological and epidemiological interest: (i) *bla*_NDM-5_ and *mcr-1* co-producing ST10 *E. coli* showed decreased fitness and virulence, compared to a ST4981 isolate harboring only *bla*_NDM-5_ isolated from the same patient (ii) acquisition of the plasmid carrying *mcr-1* or *bla*_NDM-5_ was not associated with a statistically significant fitness cost to the parent strain.

Diverse sequence types (STs) of *mcr-1*-positive strains were identified among different regions, with some commonality in clades isolated from clinical samples, such as ST10 (Wang et al., 2017). Furthermore, ST10 has also been found in asymptomatic carriage of *E. coli* co-harboring *bla*_NDM−1_ and *mcr-1* from a healthy individual (Zhong et al., 2016). To the best of our knowledge, this is the first report of ST10 in *E. coli* carrying both *bla*_NDM-5_ and *mcr-1* from clinical infections. Identification of ST10 in *bla*_NDM_- and *mcr-1*-producing *E. coli* from both hospi-talized and healthy people warrants our immediate awareness.

Exotoxins, endotoxins, adherence factors, secretion systems, iron acquisition and fimbria are commonly considered to be important virulence features in gram-negative bacteria (Cosentino et al., 2013). Type III secretion systems are associated with transport of virulence proteins across bacterial and host cell membranes into the target cells (Cornelis and Van Gijsegem, 2000). Most bacteria are reported to carry only one type III secretion system, but some isolates have two such systems, which referred as type III secretion 2 (ETT2) in *E. coli* (Yao et al., 2009). ETT2 comprises the *eiv, epr* and *epa* genes and the deletion mutant of ETT2 exhibited defects in invasion and intracellular survival (Yao et al., 2009). In this study, ETT2 was one of the potential explanations for increased virulence in GZ3 respect GZ2 *in vitro* and *in vivo*.

Although it is commonly considered that, during the absence of antibiotic pressure, resistance usually imposes a fitness cost on the bacteria (Andersson and Hughes, 2010), several controversies have arisen regarding the association of resistance and fitness. Although a considerable reduction in fitness was observed by newly acquired plasmids and other mobile genetic elements (Starikova et al., 2013; Göttig et al., 2016), some studies showed that carbapenemase-encoding plasmids contributed to low to moderate fitness cost to host (Schaufler et al., 2016; Di Luca et al., 2017). To explore whether *bla*_NDM-5_ or *mcr-1* plasmid imposed a fitness cost on the host, the growth kinetics were investigated in the transconjugants. No significant fitness cost was observed between the transconjugants and the recipient *E. coli* J53, indicating the potential dissemination of *mcr-1* and *bla*_NDM_ among *Enterobacteriaceae*.

For the isolates carrying *mcr-1* and *bla*_NDM-5_, most studies illustrated that *bla*_NDM-5_ was mediated by IncX3 plasmids (Li et al., 2016). pGZ2-NDM belonged to IncX3 in this study, but was much longer in length and carried numerous resistance genes. One explanation was that the patient suffered long course of diseases with complex intra-abdominal infections and received various kinds of antimicrobials. Therefore, the plasmid might have evolved rapidly under great selective pressures.

In previous studies, *bla*_NDM-5_ and *mcr-1*-producing *E. coli* isolated from a duck was reported in Guangzhou (Yang et al., 2016). Although the patient in this study was not engaged in animal husbandry, she might have had the chance to be in contact with poultry, because she lived in a rural area. The possibility of transmission of strains carrying *mcr-1* and *bla*_NDM_ between animals and humans remains unclear. In addition, there were some limitations in this study. No transconjugants co-harboring *mcr-1* and *bla*_NDM-5_ were obtained in this study. Thus, we have no access to determine the role of *mcr-1* and *bla*_NDM-5_ co-producing plasmids in the decreased fitness in GZ2. Further studies on biological effects of acquisition of *mcr-1* and *bla*_NDM_ plasmids are needed.

In conclusion, we reported the decreased fitness and virulence in a *mcr-1* and *bla*_NDM-5_ co-producing ST10 strain against a ST4981 *E. coli* harboring *bla*_NDM-5_ isolated from the same patient. However, no significant fitness cost was observed by carriage of the *mcr-1* or *bla*_NDM-5_ plasmid, indicating the potential for dissemination of *mcr-1* and *bla*_NDM-5_ in Enterobacteriaceae.

## Author contributions

HW conceived and designed the study. KL reviewed the medical records of the case and provided these three isolates. YZ, QW, XW, HL, and RW performed experiments described in this study. HG and YZ did the genomic analysis. YZ wrote the draft and HW revised it. All authors approved the final version.

### Conflict of interest statement

The authors declare that the research was conducted in the absence of any commercial or financial relationships that could be construed as a potential conflict of interest.
